# Filling a Gap in Developmental Toxicity Testing: Neural Crest Cells Offer Faster, Cheaper, Animal-Free Testing

**DOI:** 10.1289/ehp.120-a320b

**Published:** 2012-08-01

**Authors:** Rebecca Kessler

**Affiliations:** Rebecca Kessler, based in Providence, RI, writes about science and the environment for various publications. She is a member of the National Association of Science Writers and the Society of Environmental Journalists.

The neuronal development of fetuses and infants is exquisitely sensitive to disruption by various environmental factors. Yet few chemicals in widespread use have been thoroughly tested for developmental neurotoxicity. Most such testing relies on animal studies, a laborious and costly process that is not always a good predictor of human health outcomes. A team of researchers now describes a faster, cheaper, and more humane approach to developmental neurotoxicity testing using human neural crest (NC) cells *in vitro* [*EHP* 120(8):1116–1122; Zimmer et al.].

NC cells separate from the neural tube and migrate during embryonic development, giving rise to a wide variety of cell types that form the peripheral nervous system, bone and cartilage in the head, and other tissues. Certain drugs and environmental chemicals interfere with this migration, causing serious developmental defects.

**Figure:**
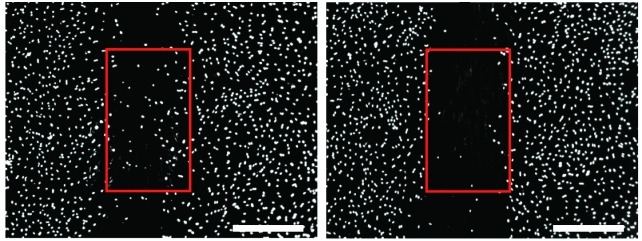
Representative images show normal (untreated) and blocked (treated) cell migration on the MINC assay. Zimmer et al.

The researchers based their new test, called the MINC (“migration of NC”) assay, on the previously established scratch assay. In this test, a gap is scratched into a monolayer of cells, and a chemical is added to measure its effect on the cells’ attempts to migrate across the gap. The researchers showed that the MINC assay detected impairment of NC cell migration by the neurotoxicants methylmercury and lead-acetate with very high sensitivity. More important, the MINC assay—but not an assay using other neural precursor cells—detected the antiepileptic drug valproic acid in the low micromolar range. Valproic acid is a human reproductive toxicant known to interfere with NC cell migration in several species.

The researchers also substituted several other types of migratory cells for NC cells in the test, but none were as sensitive to methylmercury or lead-acetate. The specificity of the test to neurotoxicants was indicated by its detection of methylmercury, lead-acetate, valproic acide, and the fungicides triadimefon and triadimenol, whereas aspirin, acetaminophen, and mannitol (a sugar alcohol used in foods and medical applications) showed no effect on NC cells—all consistent with expectations. Moreover, three forms of mercury were ranked according to their potency as disruptors of NC cell migration, suggesting that the assay may be useful in predicting the toxicity potency of a broad range of compounds.

The researchers showed that NC cells can be produced from human embryonic stem cells in large quantities, frozen, and thawed for use in the MINC assay—features that would make them easy to transport and use in laboratories around the world. They envision the MINC assay as part of a suite of cell-based tests that together could be used to quickly assess numerous potential toxicologic effects of chemical compounds.

